# Modelling of P-wave velocity changes in coal seams with increased depth: a case study

**DOI:** 10.1038/s41598-025-87417-6

**Published:** 2025-01-27

**Authors:** Maciej Łapczyński, Zenon Pilecki, Krzysztof Krawiec, Artur Słomian, Elżbieta Pilecka, Tomasz Łątka

**Affiliations:** 1 Mineral and Energy Economy Research Institute of the Polish Academy of Sciences, Wybickiego 7A, 31-261 Kraków, Poland; 2 JSW SA KWK Borynia-Zofiówka-Bzie, Aleja Jana Pawła II 4, 44-330 Jastrzębie-Zdrój, Poland; 3 Cracow University of Technology, Warszawska 24, 31-155 Kraków, Poland; 4 University of Warsaw, Krakowskie Przedmieście 26/28, 00-927 Warszawa, Poland

**Keywords:** Seismic profiling, Refracted P-wave, Coal seams, State of stress, Natural hazards, Solid Earth sciences, Engineering

## Abstract

Seismic profiling in a coal seam enables the determination of anomalous changes in the P-wave velocity compared to reference velocity at a specific mining depth, indicating potential stress changes. This information can improve the coal exploitation processes in advance at greater depths, especially in seismic hazard areas. This study aims to update the empirical mathematical formula for calculating reference P-wave velocities in coal seams by including new data measured at greater depths. The research was performed at the Zofiówka Coal Mine in the geological and mining conditions of the Upper Silesian Coal Basin in Poland. The analysis involved the study of 276 velocity values including 24 new velocity values measured at depths ranging from 704 to 1073 metres in the period 2009–2024. Through regression analysis, the standard model was modified. The new model provides more reliable velocity anomaly calculations and accurately reflects the geomechanical conditions at greater depths in the Zofiówka Coal Mine. The calculation procedure can be utilised to develop velocity models for various geological and geomechanical conditions in underground mines that exploit coal seams at continuously increasing depths.

## Introduction

Many geological and mining factors influence the development of unfavourable stress concentrations in rock mass during the exploitation of various mineral resources e.g.^1,2^. In multi-seam hard coal mining, these unfavourable stress concentrations are often caused by the tectonics in the rock mass as well as the left edges and remnants of the coal seams^3–6^. Many mathematical and empirical methods can be used to recognise such stress concentrations^7–10^, above all, there are geophysical methods^11–13^. Commonly used geophysical methods in hard coal mines include seismological^14–16^, seismoacoustic^17–21^ and seismic techniques in the form of various types of tomography and profiling^22–39^.

Seismic profiling in a coal seam enables anomalous changes in the P-wave velocity to be determined through comparison with a reference velocity. This reference velocity characterises the stress state in the rock mass at a specific depth. It mainly depends on several factors, such as gravity, properties, and the structure of the medium^40,41^. Changes in P-wave velocity that differ anomalously from the reference P-wave velocity indicate the influence of factors that may affect the exploitation technology. This information can be used to improve the coal seam exploitation processes in advance, particularly in seismic hazard areas.

The formula for calculating reference velocity in coal seams was developed by Dubiński in 1989^29^ for mining operations in the Upper Silesian Coal Basin (USCB) at depths ranging from 500 to 900 m. Velocity measurements were largely taken within this interval, with limited data from depths below 900 m, confining the formula’s initial applicability. However, current mining activities in several USCB coal mines extending below 1200 m and frequently exceeding 1000 m. In consequences, the geomechanical conditions have evolved, altering the dependence of P-wave reference velocity on depth. This necessitates a reevaluation and potential update of Dubiński’s formula to ensure its applicability under current conditions.

**Figure 1:**
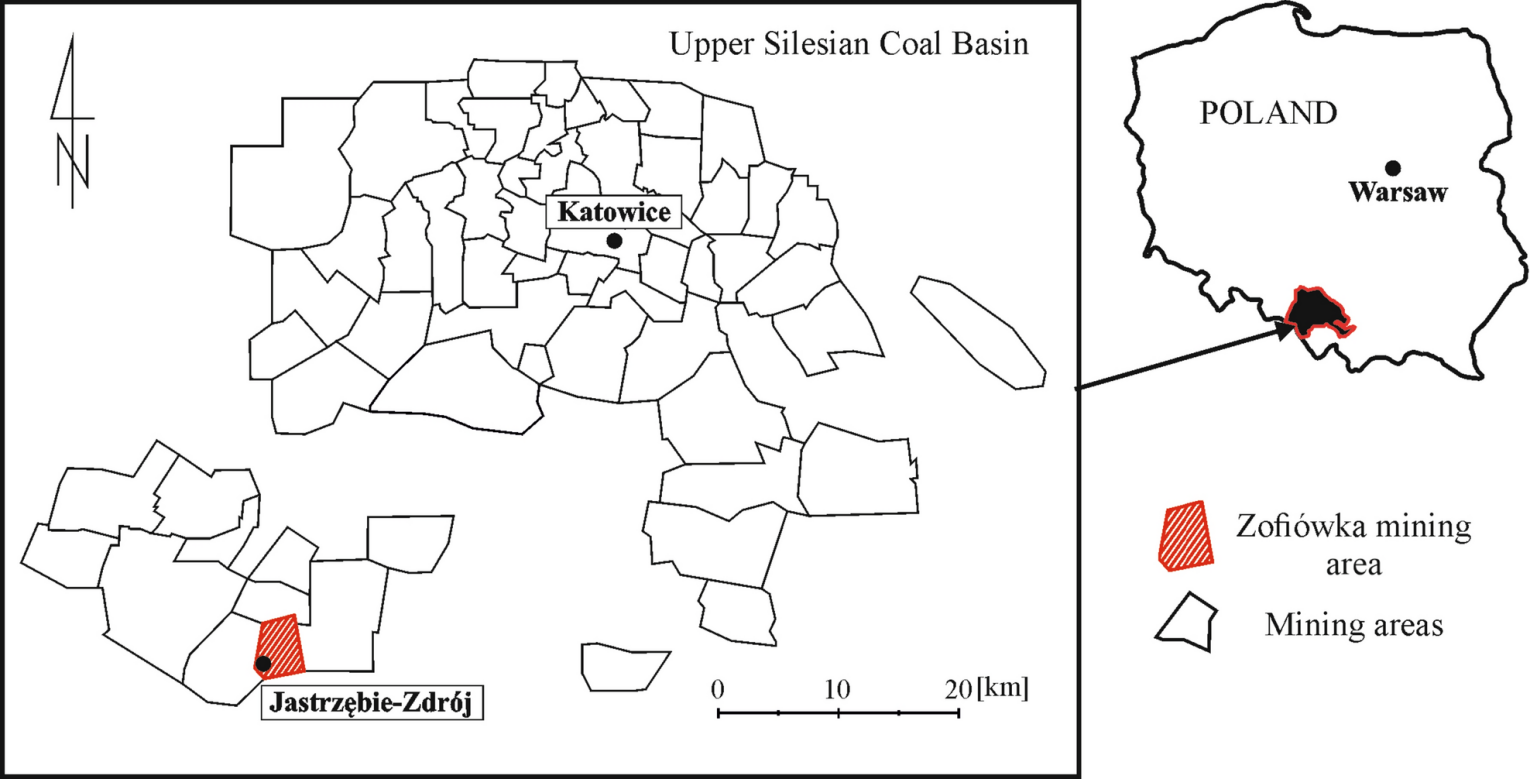
Location of the Zofiówka Coal Mine and other mining areas in the Upper Silesian Coal Basin in Poland (based on^46^).

The study aims to verify and update the empirical formula for calculating reference P-wave velocity in coal seams by including new data from greater depths. The research particularly focuses on the geological conditions of the Zofiówka Coal Mine in the Upper Silesian Coal Basin (Fig. 1).

The methodology of calculating the new coefficients of the velocity model was based on the procedure proposed by^42^ for the neighbouring Jastrzębie Coal Mine. The revised formula aims to more precisely determine seismic anomalies, reflecting the geomechanical conditions of the Zofiówka Coal Mine. Thereby it can contribute to safer and more efficient coal seam exploitation through enhanced planning of rockburst prevention measures.

Since the development of the Dubiński formula in the nineteen-eighties^29^, research methodologies have substantially advanced, as elaborated in the Theoretical Background chapter. Modern improvements in equipment and seismic data processing have significantly enhanced the efficiency of seismic research^43^.

## Theoretical background

**Figure 2:**
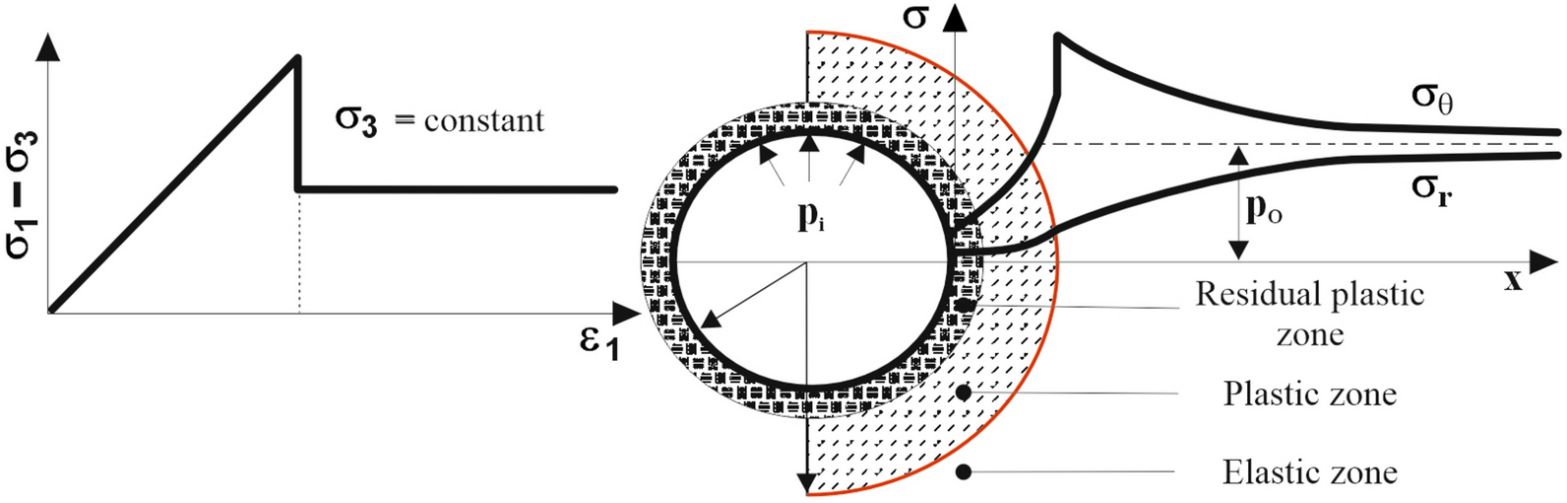
Model of the behaviour of a rock mass around an excavation (based on^44^); $\sigma _1$ - major principal stress, $\sigma _3$ - minor principal stress, $\sigma _{\theta }$ - tangential stress, $\sigma _r$ - radial stress, $\varepsilon _1$ - major principal strain, $p_0$ - virgin stress, $p_i$ - lining load.

Seismic profiling in coal seams allows the measurement of the velocity of the refracted P-wave, which propagates through undisturbed elastic zones close to the border with disturbed plastic and residual zones near excavation, the so-called excavation disturbed zone (EDZ). The location of the theoretical boundary between elastic and plastic zones is denoted by the red line in Fig. 2, using the Ladanyi model^44^.

**Figure 3:**
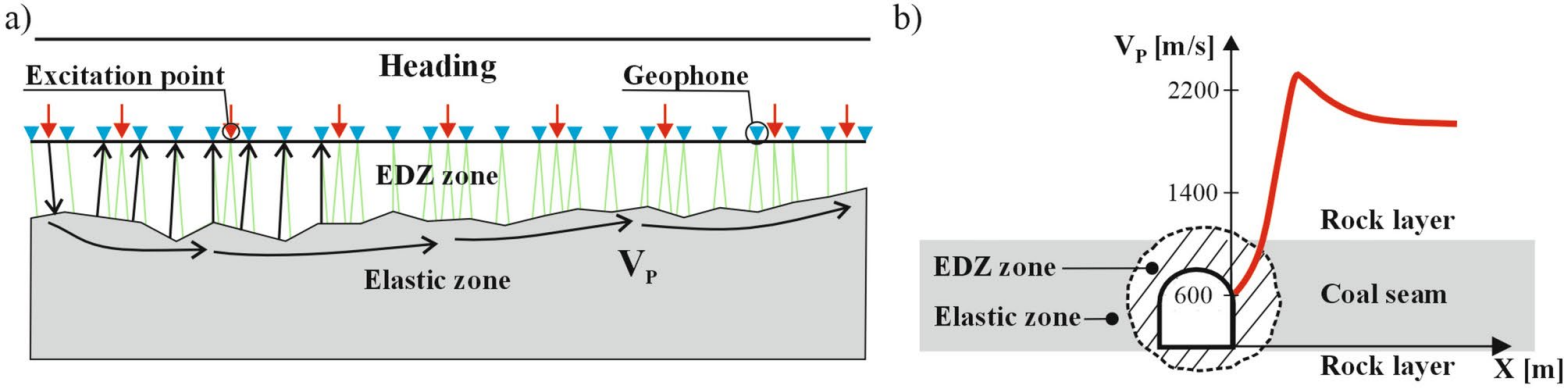
Scheme for seismic profiling to measure changes in P-wave velocity ($V_P$) in the sidewall of a heading in a coal seam (**a**); a cross section depicting the structure of a coal seam with neighbouring rock layers around the heading, highlighting the velocity of P-wave changes in the coal seam in red propagating through EDZ zone (**b**), (based on^31^).

At the border between the elastic and EDZ zones, where tangential stress reaches its highest value, changes in wave velocity correspond with stress variations. Using seismic profiling, we can determine the P-wave velocity in the coal seam along a specific section (Fig. 3). However, in field conditions, the boundary between the elastic and EDZ zones has a transitional form, resulting in a complex wave field within the structure of the coal seam and its neighbouring rock layers.

**Figure 4:**
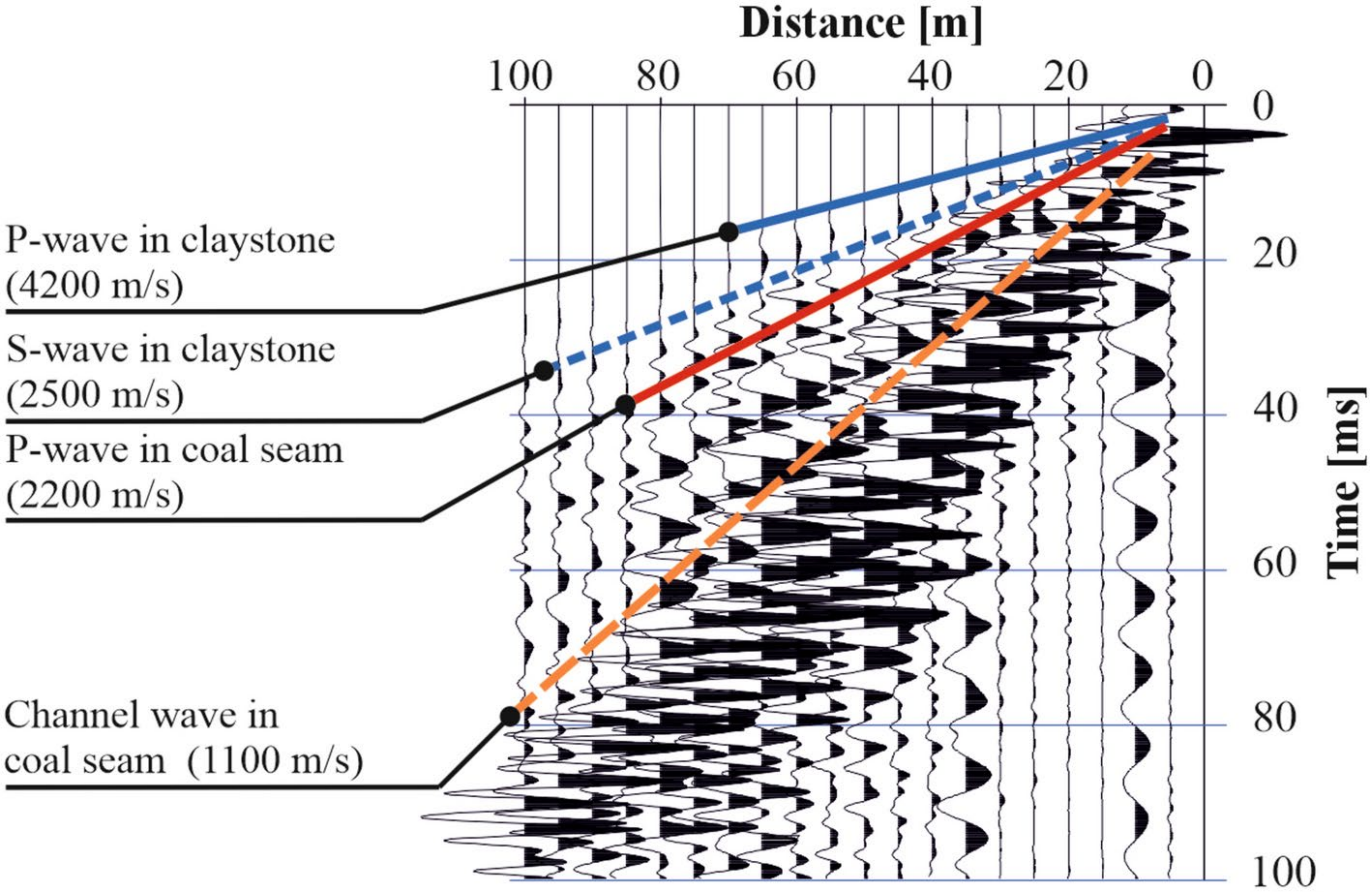
An example of a seismogram with selected waves registered in the structure of a coal seam with neighbouring rock layers.

Furthermore, identifying the first breaks of a P-wave in a heterogeneous medium is challenging due to the complex wave interactions in the EDZ zone. Under these conditions, several types of waves, including direct, reflected, diffracted, refracted waves and channel waves, can be generated. An example seismogram of the wave field is depicted in Fig. 4.

The measured $V_P$ velocity of the P-wave enables the determination of seismic anomalies in coal seams at specific mining depths. The $V_P$ velocity is compared to a $V_0$ reference velocity calculated from an empirical formula known as the Dubiński formula, given by the equation^29^:

1 $$\begin{aligned} V_0 = 1200 + 4.83 \cdot h^{0.76} \end{aligned}$$

where *h* is the depth of the measured P-wave velocity.

Finally, the seismic anomaly *A* is calculated using the formula:

2 $$\begin{aligned} A = \frac{V_P - V_0}{V_0} \cdot 100 \, [\%] \end{aligned}$$

**Figure 5:**
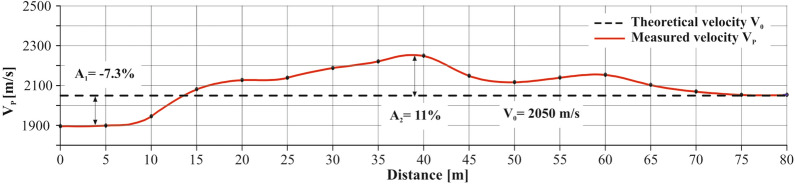
An example of calculating seismic anomaly in a coal seam at a depth of 900 m.

An example of a determining seismic anomaly is shown in Fig. 5, illustrating the practical application of this calculation in field conditions. This seismic anomaly enables the determination of the relative increase or decrease in stress within the coal seam^9^. The scale that correlates seismic anomaly values to corresponding stress changes within the USCB geological conditions for depths ranging from 500 to 900 m is detailed in Table 1.

**Table 1:** Scale of relative stress corresponding to seismic anomaly variations in the geological conditions of the Upper Silesian Coal Basin for depths from 500 to 900 m^9^.

Degree of relative stress change	Scale of relative stress increase [%]	Positive seismic anomaly [%]	Increase in relative stress [%]	Negative seismic anomaly [%]
0	Very low	Below 5	Below 20	Above 7.5
1	Low	5 to 15	20 to 60	− 7.5 to − 15
2	Medium	15 to 25	60 to 140	− 15 to − 25
3	High	Above 25	Above 140	below − 25

## Geological setting

The geological structure of the “Zofiówka” mining area deposit to a depth of 1,300 m, includes Quaternary, Neogene and Carboniferous formations.

Quaternary deposits cover the entire surface of the mining area and range in thickness from around 10 m to around 90 m. This formation involves a large area of permeable sand and gravel deposits covered with silty clay. The thickness of the clays ranges from 2 m to 20 m.

The Neogene deposits are mainly grey-green clays and are sometimes dusty or sandy. They range in thickness from 196 m to 873 m.

The Carboniferous formations are represented by Orzesze, Ruda and Saddle layers (Table 2). The Orzesze layers contain coal seams ranging from 346/1 to 363. Lithologically, they are formed as claystones with varying degrees of sandiness, mudstones with coal seams and sandstones, which are most often fine-grained, with clayey and clay-silica binders. Coal seams most often occur among clay shales and, to a lesser extent, in contact with sandstones in the roof. The Ruda and Orzeskie layers are formed in clay-stone-sandstone-mudstone facies with sparse coal seams and thin sandstone layers. The Upper Ruda layers contain coal seams from 401 to 407/3. However, the Lower Ruda layers have thicker sandstone layers up to several metres high; they are medium- and coarse-grained, and over thirty coal seams have greater thicknesses. The Saddle layers contain coal seams ranging from 501/1 to 510. Lithologically, the Saddle layers are formed as medium- and coarse-grained thick-bedded sandstones, often different-grained or conglomeratic with claystone inserts accompanying coal seams.

Regarding tectonic structure, the “Zofiówka” deposit can be divided into a western part and an eastern part, and the boundary of this division is the “Jastrzębski” fault. In the western part, the layers dip monoclinally to the east at an angle of $10\text {-}15^{\circ }$, and the extension of the layers is close to the N-S direction. In the eastern and south-eastern parts, the deposit dips more gently at an angle of $2\text {-}7^{\circ }$, with the general extension of the layers being NW-SE. A dense network of normal faults cuts the Carboniferous formation, less frequently reverse faults, with throws ranging from several centimetres to over 60 metres. The latitudinal and meridional directions dominate.

**Table 2:** Coal seams in the Zofiówka mining area in a fragment of lithostratigraphic units of the Upper Silesian Coal Basin (based on^45^).

Stratigraphy	Lithology	Local stratigraphy		Coal seam numbers	Zofiówka coal seams
Pennsylvanian	Krakow Sandstone	Libiąż		111–119	
		Łaziska		201–216	
	Mudstone	Orzesze*	Orzesze	301–327	
			Załęże	328 - 364	346–510
		Ruda*		401–406	
	Upper Silesian Sandstone		Ruda	407–420	
		Zabrze (Saddle, Anticlinal)		501–510	

*Former mining subdivision

## Methods and data

The calculation method is similar to the procedure in^42^ and involves three main stages:

I. Preparation of new data from seismic profiling in coal seams at greater depths in the Zofiówka Coal Mine. This involves creating a dataset that includes both new and archived values.

II. Analysing the P-wave velocity-depth relationship for the combined data using linear and power-law models.

III. Verification of statistical models, selection of the best fitting model and calculation of systematic errors.

In the first stage, P-wave velocity values were determined from seismic profiles in sections unaffected by mining and geological factors such as coal seams edges, remnants and faults. Measurements were taken at depths ranging from 704 to 1073 metres in various coal seams, including 407/1, 409/3, 409/4, 409/9, 410, 412, 416/3, 417/1, 502/1 and 505/1. A total of twenty-four new P-wave velocity values were collected from measurements taken between 2009 and 2024 (Table 3).

**Table 3:** Zofiówka Coal Mine—new values of P-wave velocity.

No.	Depth [m]	Measured velocity of the P-wave [m/s]	No.	Depth [m]	Measured velocity of the P-wave [m/s]
1	704.0	1920	13	983.4	2298
2	744.8	2011	14	987.3	2101
3	883.7	2106	15	1015.9	2330
4	890.0	2168	16	1028.5	2312
5	906.8	2054	17	1028.8	2141
6	911.1	2196	18	1032.0	2180
7	925.7	1982	19	1053.2	2223
8	926.5	2239	20	1059.0	2140
9	935.5	2209	21	1059.0	2310
10	944.0	2159	22	1064.5	2266
11	950.0	2133	23	1068.4	2194
12	950.9	2080	24	1073.0	2208

**Figure 6:**
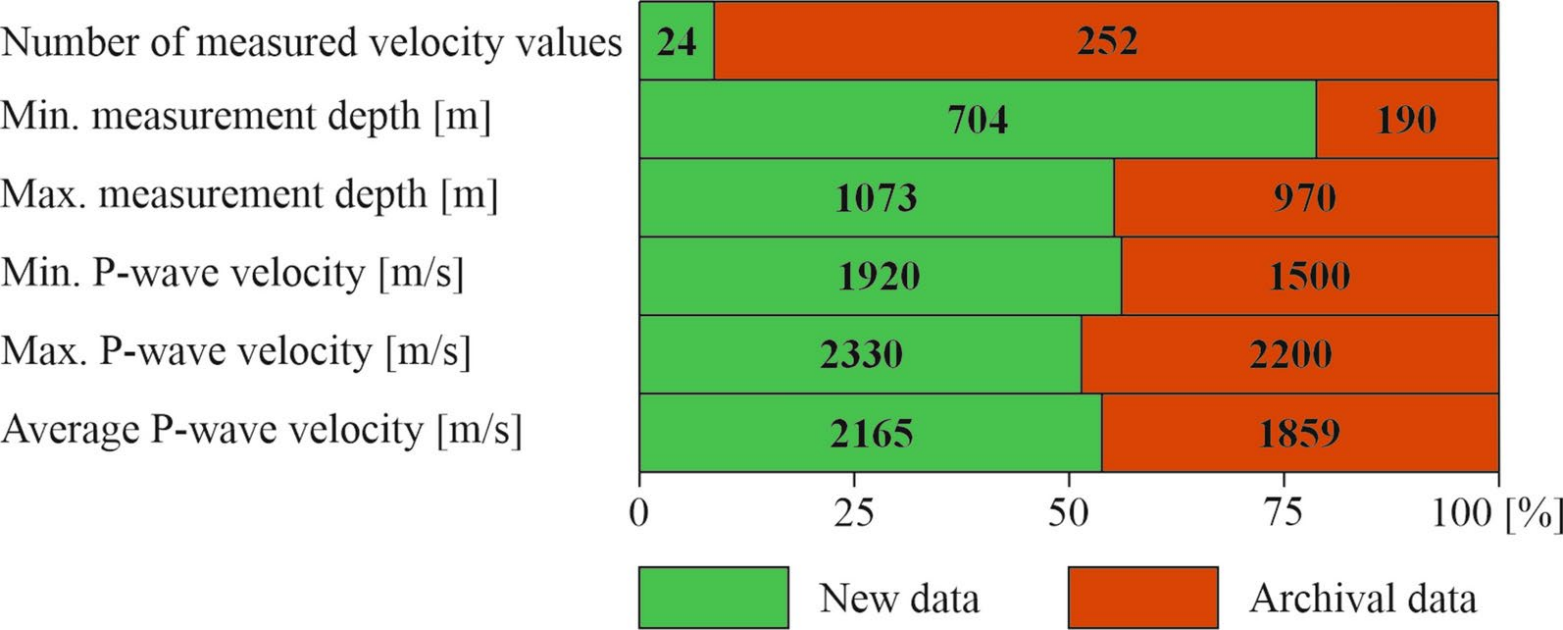
Statistics of the calculation data.

A dataset containing new and archived P-wave velocity values was prepared. The combined dataset consists of 276 values. The archived data covers the velocity range from 1500 to 2200 m/s, with an average value of 1859 m/s, while the new data from the Zofiówka Coal Mine ranges between 1920 and 2330 m/s, with an average value of 2165 m/s. The statistics of this set are presented in Fig. 6.

In the second stage, reference velocity values $V_0$ were assumed to be a function of depth $h$, according to the power model proposed by^29^: ${V_0} = 1200 + a h^b$. A linear model was also tested. Linear and nonlinear fit curves were generated for the combined data set using Statistica software. The Levenberg-Marquardt estimation method was utilised:

3 $$\begin{aligned} \Delta p = (J^T J + \lambda I)^{-1} \cdot J^T r \end{aligned}$$

where $\Delta p$ is the parameter update vector, $J$ is the Jacobian matrix of partial derivatives of the residuals with respect to the parameters, $J^T$ is the transpose of the Jacobian matrix, $\lambda$ is the damping parameter that controls the transition between the Gauss-Newton method and the gradient descent method, $I$ is the identity matrix, $r$ is the residual vector, which is the difference between the observed and model-predicted values.

The calculations used velocity values that were less than double the standard deviation $\sigma$:

4 $$\begin{aligned} \sigma = \sqrt{\frac{\sum _{i=1}^{n} \left( y_i - \widehat{y_i} \right) ^2}{n}} \end{aligned}$$

where $\sigma$ is the standard deviation, $y_i$ is the individual observed value of the dependent variable, $\widehat{y_i}$ is the value predicted by the regression model for the $i$-th observation, and $n$ is the number of observations.

An evaluation of the fit in the regression analysis was performed using the $R^2$ coefficient of determination:

5 $$\begin{aligned} R^2 = \frac{\sum _{i=1}^{n} \left( \widehat{y_i} - \underline{y} \right) ^2}{\sum _{i=1}^{n} \left( y_i - \underline{y} \right) ^2} \end{aligned}$$

where *y* is the arithmetic mean of all *y* values.

In the third stage of work, the most significant regression model for estimating P-wave velocity versus depth was verified and selected. The differences between the observed and predicted values by Root Mean Square Error (*RMSE*) and the performance of a regression model by Variance Accounted For (*VAF*) and the total systematic error were calculated. Once the best curve-fitting model was selected, it was statistically verified to check the significance of its fit.

The *RMSE* was calculated as the root mean of the squared errors:

6 $$\begin{aligned} \text {RMSE} = \sqrt{\frac{1}{n} \sum _{i=1}^{n} \left( y_i - \widehat{y_i} \right) ^2} \end{aligned}$$

*VAF* was calculated as the ratio of the total variance in the actual values that is accounted for by the variance in the predicted values:

7 $$\begin{aligned} \text {VAF} = \left( 1 - \frac{\text {Var}(y - \widehat{y_i})}{\text {Var}(y)} \right) \times 100\% \end{aligned}$$

The analysis focused on calculating the total systematic error. This included considering the uncertainty in determining the P-wave onset time on the seismogram and the location of the geophone position at the excavation sidewall. The value of this error was calculated using a total differential equation:

8 $$\begin{aligned} \left| \Delta y_r \right| = \sum _{i=1}^{n} \left| f_{x_i}^\prime \left( x_1, x_2, \ldots , x_n \right) \Delta x_i \right| \end{aligned}$$

where

9 $$\begin{aligned} \sum _{i=1}^{n} \left| f_{x_i}^\prime \left( x_1, x_2, \ldots , x_n \right) \Delta x_i \right| = \left( \left| \frac{\partial }{\partial x_1} \Delta x_1 \right| + \left| \frac{\partial }{\partial x_2} \Delta x_2 \right| + \ldots + \left| \frac{\partial }{\partial x_n} \Delta x_n \right| \right) f \left( x_1, x_2, \ldots , x_n \right) \end{aligned}$$

$y_r$ is the value of the function after changing its arguments, $x_1, x_2, \ldots , x_n$ are the independent variables, and $x_i$ is the $i$-th independent variable.

In order to assess the statistical significance of the chosen model, we calculated its basic statistics, such as the standard error and the lower and upper confidence intervals at a 95% confidence level. Furthermore, we conducted the Breusch-Pagan test to check for heteroscedasticity and created histograms of the standardised residuals before and after removing the outliers. We also created a normal probability plot of the residuals to show the data distribution after the outliers were removed.

## Results and discussion

**Figure 7:**
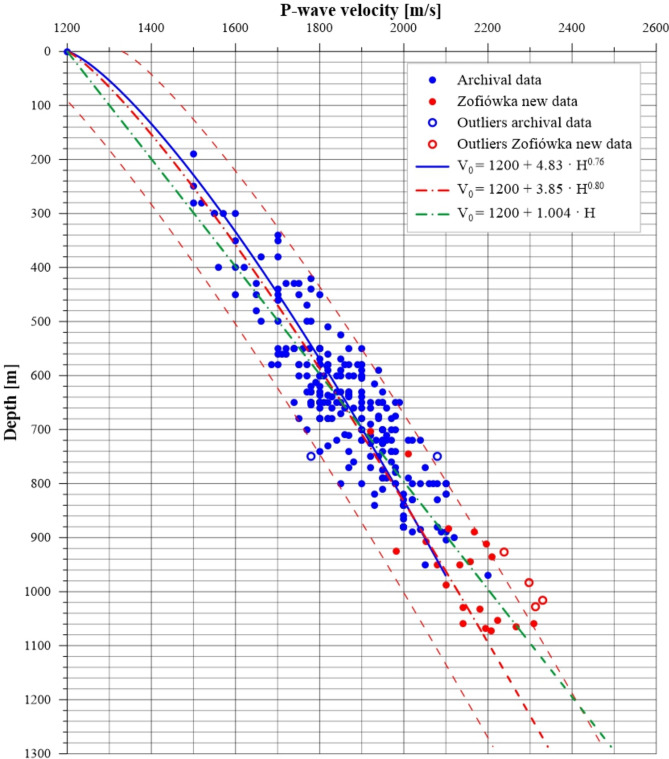
Archival data set (blue dots) and new data from the Zofiówka Coal Mine (red dots), along with the regression curve for all data: linear (green blue dash-dot line) and nonlinear (red dash-dot line) equation and the archival data (blue line). Outliers are highlighted with empty dots.

The calculation data, including archival and new data from the Zofiówka Coal Mine, is presented in Fig. 7. It is evident that the new data provides valuable information on P-wave velocity from deeper depths compared to the archived set. Assuming the approximation of the data with a power-law model, from the set of all 276 P-wave velocity measurements, six extreme values that did not meet the double standard deviation criterion were rejected. Four of these points came from the new data and two were from the archived data.

Following the rejection, calculations were performed on a set of 270 points, resulting in the following relationship for the reference velocity for the P wave vs. depth:

10 $$\begin{aligned} V_0 = 1200 + 3.85 \cdot h^{0.80} \end{aligned}$$

**Figure 8:**
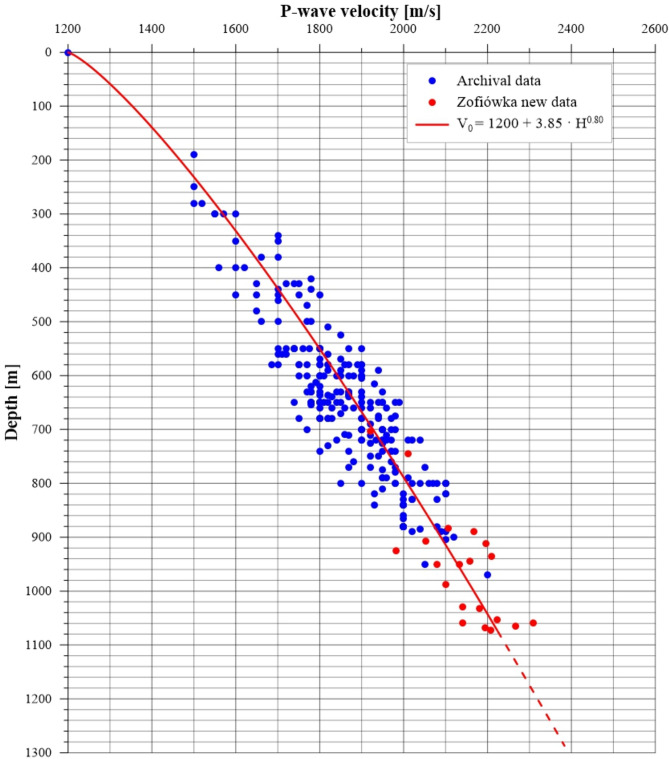
Archival data set (blue dots) and recent data from the Zofiówka coal mine (red dots) without outliers, along with the regression curve for all data (red line).

Relationship (Eq. 10) is furthermore clearly illustrated in Fig. 8. Additionally, a linear relationship was calculated in the form:

11 $$\begin{aligned} V_0 = 1200 + 1.004 \cdot h \end{aligned}$$

The coefficients *a* and *b* for Eq. 10) are quite similar to the coefficients of the formula given by^42^ for the neighbouring Jastrzębie Coal Mine (Table 4). Moreover, the coefficient of determination and standard deviation values are similar. It should be emphasized that both the Zofiówka and Jastrzębie mines operate in similar geological conditions. Table 4 presents a comparison of all formulas and their descriptive statistics. Both regression curves are characterised by the same coefficient of determination $R^2$, equal to 0.83. The $R^2$ value indicates a good fit of the model to the data set. The standard deviation of both models is 62 m/s. Considering the current form of relationship (Eq. 1), the power model (Eq. 10) was adopted for further analysis.

**Table 4:** Reference velocity $V_P$ models.

Name of formula	Reference velocity $V_P$ model	Coefficient	Standard
		of determination $R^2$	deviation $\sigma$
Dubiński’s formula	$V_0 = 1200 + 4.83 \cdot h^{0.76}$	0.77	64
Jastrzębie Coal Mine formula	$V_0 = 1200 + 2.73 \cdot h^{0.85}$	0.82	63
Zofiówka Coal Mine formula	$V_0 = 1200 + 3.85 \cdot h^{0.80}$	0.83	62

The nonlinear regression analysis was conducted with a significance level $\alpha = 0.05$. The parameter estimates are summarised in Table 5. The estimates for parameters $a$ and $b$ are 3.85 and 0.80, respectively. For parameter $a$, the standard error is 0.61 and the 95% confidence interval is $[2.65, 5.06]$. For parameter $b$, the standard error is 0.02 and the 95% confidence interval is $[0.75, 0.84]$. Both confidence intervals do not include zero, indicating the accuracy of the estimation.

**Table 5:** Estimated parameters *a*, *b* for Dubiński formula and their statistics.

Estimated parameter	Value of estimated parameter	Standard error	Lower confidence limit	Upper confidence limit
a	3.85	0.61	2.65	5.06
b	0.80	0.02	0.75	0.84

The results of the Breusch-Pagan test for heteroscedasticity in the residuals indicate no evidence of its presence, as confirmed by a probability value *p* = 0.598 from the Lagrange Multiplier test. This outcome suggests that there are no statistically significant grounds to reject the null hypothesis of homoscedasticity, implying that the variance of the residuals is constant. This supports the conclusion that the model may be adequate for the data with respect to one of the key assumptions of regression. However, a complete assessment of the model’s validity would require further tests that cover the remaining assumptions.

**Figure 9:**
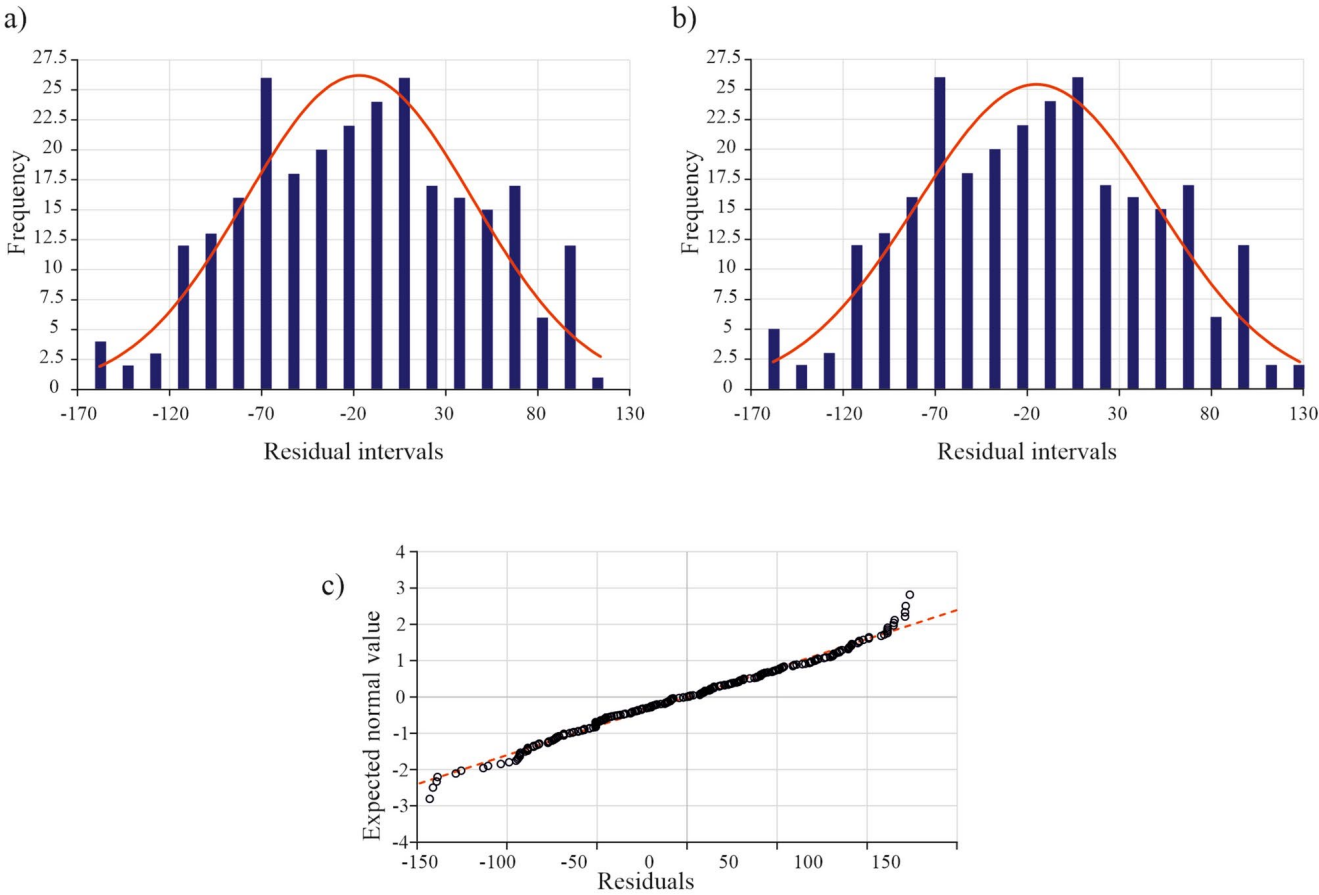
(**a**) Histogram of standard residuals (dark blue bars) and density of normal distribution plot (red line) with outliers, (**b**) without outliers, (**c**) normal probability plot of residuals (black circles) along with regression curve (red dashed line).

To assess the normality of the data, we examined standardised residuals by generating histograms before and after the removal of outliers. The histograms reveal that the residuals follow a bell-shaped curve both before (Fig. 9a) and after correction (Fig. 9b), which is consistent with a Gaussian distribution.

By analysing the normal probability plot of the residuals (Fig. 9c), it is evident that most points closely follow the trend line, suggesting that the distribution of residuals may be close to normal. While there are some deviations at the extremes of the plot, they are not significant, indicating minor anomalies in the distribution of residuals that do not substantially impact the results and statistical inference.

Consequently, the residuals have a distribution that is close to normal, which is desirable in regression analysis. Histograms of the standardised residuals also confirm their normal distribution, thereby enhancing the credibility of the model, supporting its validity, and ensuring the reliability of error estimates and calculated coefficients.

The seismic profiling measurements are affected by two main systematic errors: (1) determining the time of arrival of the P wave in the seismogram with an error of 0.125 ms and (2) a geophone location error of 0.20 m. The maximum systematic error can be calculated using the formula:

12 $$\begin{aligned} \Delta V_P = \left| \frac{\partial V_P}{\partial d} \Delta d \right| + \left| \frac{\partial V_P}{\partial t} \Delta t \right| = 20\ \text {m/s} + 25\ \text {m/s} = 45\ \text {m/s} \end{aligned}$$

where $V_P$ is the P-wave velocity, $d$ is the length of the seismic profile, and $t$ is the arrival time of the P-wave.

The calculated maximum systematic error of 45 m/s is comparable to the difference in the estimated velocity using the standard Eq. (1). For example, for a depth of 1000 m, the difference is 47 m/s. This means that the new formula for the Zofiówka Coal Mine enables the calculation of the reference velocity in a slightly different way from the maximum systematic error using the standard formula.

The calculated *RMSE* and *VAF* indicators were $61\ \text {m/s}$ and $83.27\%$, respectively. These indicators demonstrate that the model can adequately fit the data and effectively calculate the P-wave reference velocity in coal seams at the Zofiówka Coal Mine.

## Conclusions

Changes in geomechanical conditions at greater depths in the Zofiówka Coal Mine have led to adjustments in the standard P-wave reference velocity-depth relationship. A statistical analysis was conducted, leading to the following conclusions:

1. A new power model in $V_0 = 1200 + 3.85 \cdot h^{0.80}$ was proposed, allowing a more accurate estimation of the reference velocities in calculating the seismic anomaly. The analysis was based on 276 P-wave velocity values, including twenty-four new velocity values in the depth range from 704 to 1073 m.

2. The proposed power model fits the data well after rejecting six outliers. The coefficient of determination $R^2 = 0.83$, the Root Mean Square Error $\textit{RMSE} = 61 \, \text {m/s}$, and the Variance Account For $\textit{VAF} = 83.3\%$. The standard residuals indicated a nearly normal distribution. The residuals also exhibited a good correlation with the trend line, with minor deviations at the ends of the distribution. The Breusch-Pagan test did not show heteroskedasticity (*p* = 0.598), indicating the stability of the residual variances.

3. The coefficients of the new model do not significantly differ from the standard model and are similar to those obtained for the neighbouring Jastrzębie Coal Mine. This validates the accuracy of the models calculated under similar geological conditions for the Zofiówka and Jastrzębie mines.

4. With ongoing exploitation at greater depths, there is a need for periodic updates to the formula for calculating reference velocity.

The proposed research procedure can be applied to other geological conditions to improve the safety of mining activities at greater depths by applying more precise rock-burst prevention measures.

## Data Availability

The datasets used and/or analysed during the current study are available from the corresponding author upon reasonable request.
